# Acute Self-Induced Poisoning With Sodium Ferrocyanide and Methanol Treated With Plasmapheresis and Continuous Renal Replacement Therapy Successfully: A Case Report

**DOI:** 10.1097/MD.0000000000000890

**Published:** 2015-05-29

**Authors:** Zhenning Liu, Mingli Sun, Hongyu Zhao, Min Zhao

**Affiliations:** From the Department of Emergency Medicine, Shengjing Hospital of China Medical University (ZL, HZ, MZ); and Pharmacy College, China Medical University, Shenyang, China (MS).

## Abstract

Self-induced poisoning with chemicals is one of the most commonly used suicide methods. Suicide attempts using massive pure sodium ferrocyanide and methanol are rare. This article discusses the management of acute intentional self-poisoning using sodium ferrocyanide and methanol.

We present a case of acute self-induced poisoning using sodium ferrocyanide and methanol admitted to our hospital 2 hours after ingestion. He was deeply unconscious and unresponsive to painful stimuli. The laboratory findings showed acute kidney injury and severe metabolic acidosis. We took effective measures including endotracheal intubation and mechanical ventilation to ensure the vital signs were stable. Subsequently, we treated the patient using gastric lavage, bicarbonate, ethanol, plasmapheresis (plasma exchange), and continuous renal replacement therapy (CRRT) successfully. He gradually recovered from poisoning and was discharged without abnormalities on the 6th day. Follow-up for 3 months revealed no sequelae.

Blood purification including plasmapheresis and CRRT is an effective method to scavenge toxicants from the body for acute self-poisoning with sodium ferrocyanide and methanol. Treatment strategies in the management of poisoning, multiple factors including the removal efficiency of toxin, the protection of vital organs, and the maintenance of homeostasis must be considered.

## INTRODUCTION

Around 800,000 to 1 million people die by suicide every year, making it one of the leading causes of death in the world.^1^ Suicide by self-poisoning is a major cause of death worldwide. Suicide attempts using massive pure sodium ferrocyanide and methanol were rare in the previous literature. Methanol intoxications can cause severe metabolic acidosis, exhaustion of ATP (adenosine triphosphate), visual disturbances, and neurological deficit. The metabolism of methanol is responsible for the transformation of methanol to its toxic metabolites, especially formic acid.^2^ The minimal poisoning dose of methanol in humans has been assumed to be 100 mg/kg body weight and about 30 mL of pure methanol may cause death. Sodium ferrocyanide is one of the commonly used food additives (acceptable daily intake 0–0.025 mg/kg body weight) with a low toxicity. However, if the 2 chemicals were orally ingested, their toxic effects would be significantly severe.

## CASE REPORT

### Patient Information

A 24-year-old male patient weighing 80 kg was admitted to the emergency department with his parents. He was a student majoring in chemical industry research. He had been emotionally unstable due to a breakup, and hence was deeply depressed. He did not consult any psychiatrists at this point. He was found unconscious in an isolated room for about 2 hours, and 2 bottles filled with methanol and sodium ferrocyanide from his laboratory were also found in his room. We considered he might have orally ingested about 100-mL methanol and 50-g sodium ferrocyanide according to the remnants of the chemicals.

### Clinical Findings

Physical examination revealed blood pressure of 78/34 mmHg, heart rate of 56 beats/minute, and irregular respiratory rate. He was in deep coma (Glasgow score 10) with poor peripheral circulation and pulse oxygen saturation of 65%.

### Diagnostic Assessment

After he was admitted to the emergency department, blood tests and toxicant analysis were carried out promptly. The laboratory data presented in Table 1 showed increased leukocytes, respiratory failure, acute kidney injury, and metabolic acidosis. Additionally, the levels of sodium ferrocyanide, methanol, and its product formic acid at 2 hours after ingestion were 361.2, 1244.1, and 728.6 mg/L, respectively. According to these results, we confirmed he was suffering from methanol and sodium ferrocyanide poisoning.

**TABLE 1:**
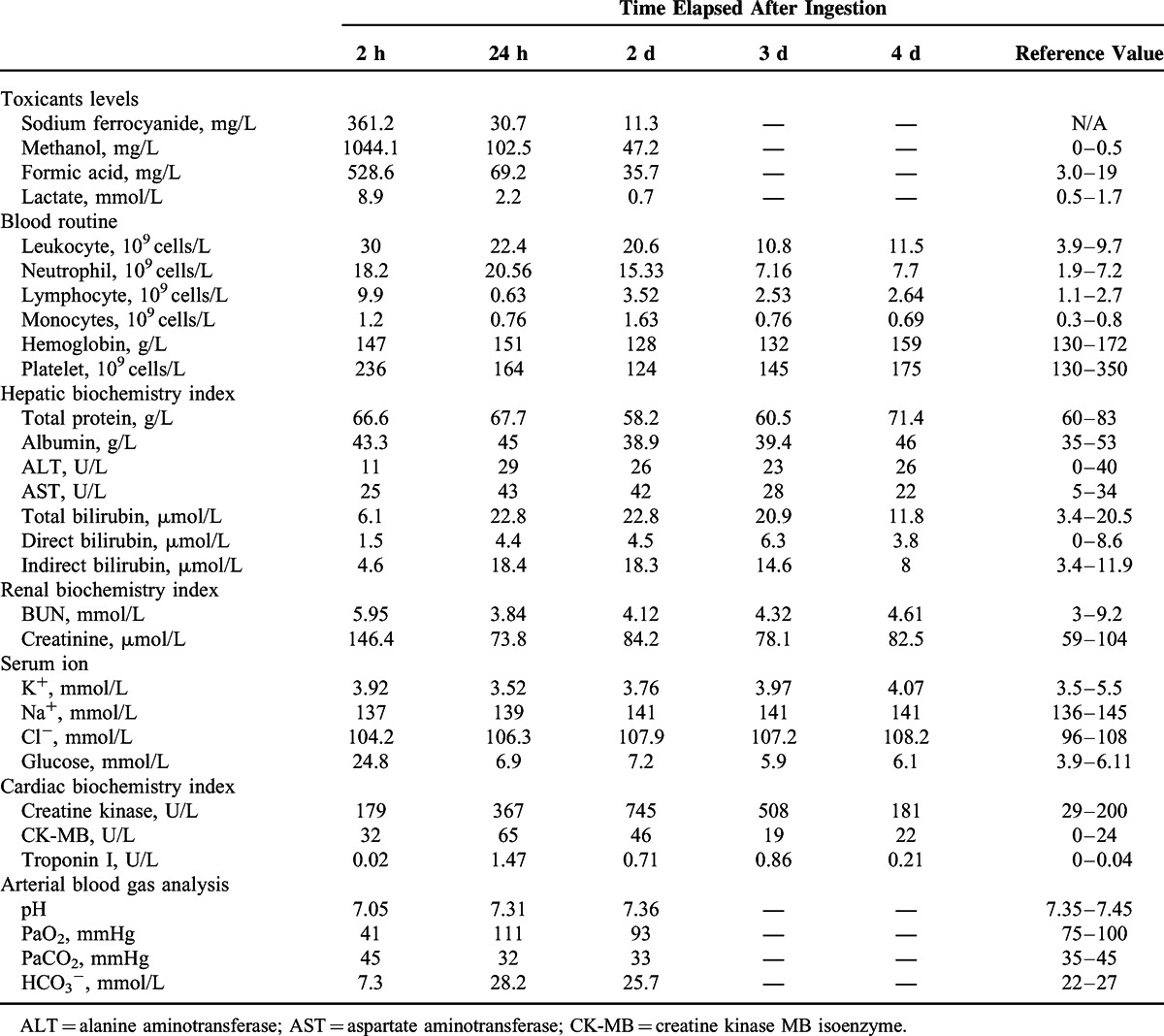
Toxicants Levels in Serum and Basic Laboratory Test

### Therapeutic Intervention

**FIGURE 1:**
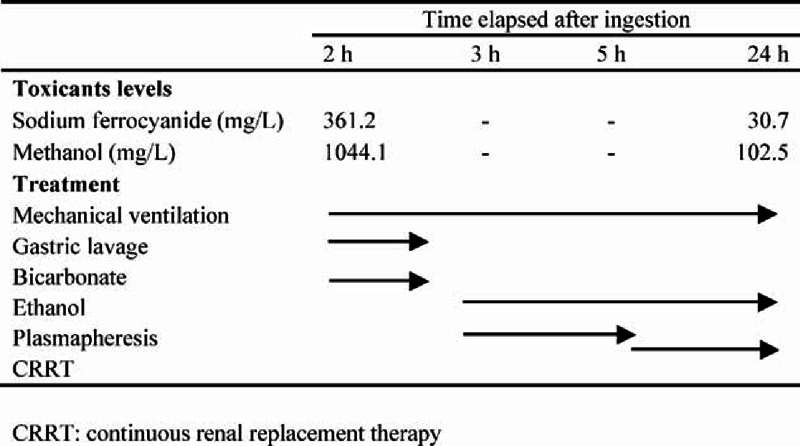
Toxicants levels in serum and main treatment details according to time elapsed after ingestion.

Endotracheal intubation, mechanical ventilation, gastric lavage, and other essential supportive therapies were used immediately. Meanwhile, he was treated intravenously with 5% sodium bicarbonate (150 mL) to manage metabolic acidosis based on the arterial blood gas analysis results. After gastric lavage, the ethanol was administered as a bolus loading with 120 mL of 40% ethanol (0.6 g/kg) followed by a maintenance dose of 0.2 g/kg/h through the indwelling nasogastric tube. At 3 hours after ingestion, a single plasmapheresis was initiated. During the 2-hour therapy, 3000-mL replacement solutions including 2000-mL fresh frozen plasma and 1000-mL normal saline solution were administered, and 2400-mL plasma was removed from the patient. After plasma exchange, the patient had to undergo continuous veno-venous hemodiafiltration with a Prismaflex dialyzer (Gambro Lundia AB, Lund, Sweden) and AN69 membrane filters (M150) in post-dilution mode immediately. The flow rates of blood, PT. fluid removal, replace solution, dialysate were set for 150 mL/min, 200 mL/h, 1000 mL/h, and 1000 mL/h, respectively. The actual ultrafiltration rate and total dialysis dose (effluent rate) were 1200 and 2200 mL/h (27.5 mL/kg/h). Besides, the dialysate containing 35 mmol/L bicarbonate was used in the process. The toxicant levels were detected at 24 hours after ingestion. Both continuous renal replacement therapy (CRRT) and ethanol administration were terminated at that time due to the improved indexes. The toxicants levels in serum and main treatment details according to time elapsed after ingestion were shown in Figure 1. Compared with the initial toxicants levels, these indexes at the end of therapy including plasmapheresis and CRRT were significantly reduced. As his health was well-controlled and gradually improved, mechanical ventilation was also terminated at 24 hours after ingestion. Then, the other supportive therapies were carried out continuously.

### Follow-Up and Outcomes

At the 6th day, he was discharged from the hospital after physical recovery and received psychological treatments in a psychological clinic. He was fully recovered with no sequelae at his 3-month follow-up.

## DISCUSSION

Methanol poisoning is a lethal form of poisoning associated with serious complications including metabolic acidosis, acute kidney injury, retinal damage with blindness, and neurologic dysfunction.^3^ Methanol can be rapidly absorbed and can reach its peak values within 30 to 60 minutes. The serum half-life ranges from 14 to 30 hours and is distributed freely.^2^ Methanol is metabolized by alcohol dehydrogenase and formaldehyde dehydrogenase to formic acid, which can inhibit the function of mitochondrial cytochrome C oxidase and reduce ATP production. ATP reduction can cause multiple organ dysfunction and cell death, especially for neurons and glial cells.^4^ Lactate is produced as formic acid interferes with intracellular respiration and promotes anaerobic metabolism. Both the formic acid and lactic acid contribute to metabolic acidosis. It was confirmed that the morbidity and mortality of methanol poisoning were associated with the formic acid concentrations in serum.^5^

Sodium ferrocyanide has a low toxicity, as its LD_50_ (50% lethal dose) in rats is 1600 to 3200 mg/kg body weight when administered orally. After absorption into the bloodstream, ferrocyanide appears to be eliminated via the glomeruli and not excreted via renal tubules.^6^ The large quantities of ferrocyanide could induce acute renal failure according to the previous report.^7^ Sodium ferrocyanide can be decomposed to cyanide with high toxicity under the influence of strong acids. The dissociation constant for hydrolysis of [Fe(CN)_6_]^4−^ to Fe^2+^ and CN^−^ at physiologic pH is 10^−35^ mmol.^8^ So, it is stable in physiological condition and the cyanide release from ferrocyanide could be theoretically very low. Although some studies in vivo have demonstrated cyanide release after oral administration, the interpretation of the results should take into account the hydroxocobalamin administration, the delayed analysis,^9^ and the release of hydrocyanic acid from cyanide derivative during the gas diffusion step.^10^ Indeed, the blood cyanide levels depend on early sampling and careful storage because of the instability of cyanide in blood and the vulnerability of cyanide assays to multiple sources of interference.^9,10^ Based on the procedure that determination of cyanide is accomplished by the derivatization reaction into 1-cyano-2-benzoisoindole derivate, we used a Ultrafast Liquid Chromatography assay (detection limit is 0.5 ng/mL) to detect the blood cyanide levels in the patient. However, there were no positive results in his blood.

The management of methanol poisoning includes gastrointestinal decontamination, supportive care, the correction of metabolic abnormalities, the administration of folinic acid, the provision of an antidote (ethanol/fomepizole) to inhibit the metabolism, and hemodialysis to correct severe metabolic acidosis and to enhance methanol and formate elimination.^2^ Both ethanol and fomepizole can inhibit the metabolism of methanol to formate by inhibiting alcohol dehydrogenase activity. Once the diagnosis has been determined, ethanol and fomepizole should be administered soon after methanol exposure. Extracorporeal removal of poisons is occasionally indicated in the management of intoxications. According to the AACT/EAPCCT methanol guidelines, hemodialysis may be considered if one of the following indications is present: The amount of pure methanol ingested orally >30 mL; methanol concentration in serum >15.6 mmol/L (500 mg/L), or formic acid concentration in serum >4.34 mmol/L (200 mg/L); metabolic acidosis; optic nerve disorders; impaired consciousness.^2^ CRRT is an alternative for methanol and formic acid removal in methanol poisoning, especially for these unstable patients who truly cannot tolerate hemodialysis.^11^ Currently, there were no reports on CRRT in the treatment of ferrocyanide poisoning, but CRRT may be theoretically possible for the ferrocyanide removal, considering the water-solubility and the molecular weight (300 Da) of sodium ferrocyanide.

Owing to the advantage of hemodynamic stability and high solute clearance, CRRT is attractive for the management of poisoning. However, the distinct drawback of CRRT is the relatively slower clearance rates for toxicants poisoning compared with conventional hemodialysis.^12^ Plasmapheresis can remove toxins of all sizes, including protein- and lipid-bound toxins. In addition, plasmapheresis with fresh frozen plasma can be used to replenish the plasma components with biological activity, and eliminate inflammatory mediators from the body quickly. The effectiveness of plasmapheresis in toxins removal is determined by a variety of factors including the time between dose administration and plasmapheresis initiation, protein binding, and volume of distribution. The rapidity of plasmapheresis toxins removal is better than that of hemodialysis, peritoneal dialysis, and hemoperfusion.^13^ Therefore, plasmapheresis can compensate for the drawback of CRRT mentioned above.

In this patient, the conventional treatments including mechanical ventilation, gastric lavage and sodium bicarbonate were performed immediately after admission. Because both the intravenous ethanol and fomepizole could not be obtained in China, the oral ethanol was administered through the indwelling nasogastric tube. Considering the severity of the disease and hemodynamic instability, conventional hemodialysis was not initially considered. We used plasmapheresis first and CRRT subsequently to eliminate the chemicals and their toxic products. Unfortunately, we did not re-measure the toxin levels between plasmapheresis and CRRT to determine their respective functions. Theoretically, both methanol and sodium ferrocyanide are water-soluble compounds with small molecular weight, and their volumes of distribution are 0.5–0.8 and 0.2 L/kg, respectively. Therefore, in a patient weighing 80 kg, the volumes of distribution of methanol and ferrocyanide volume are 40 to 64 and 16 L, respectively. During plasmapheresis, the removal and replacement of 3 L plasma may have decreased methanol and ferrocyanide concentrations by 10% and 20% over the 2-hour session, respectively. It should be recognized that CRRT may play a major role in the toxins removal. However, it cannot be ignored that plasmapheresis improved the internal environment and quickly replenished the active plasma components while simultaneously removing the toxins. Plasmapheresis can pave the way for the following CRRT. The combination of plasmapheresis and CRRT may exert a beneficial effect.

In addition, serious psychological instabilities seemed to have preceded and steered the incident. In this case, although he did not receive any psychological therapy before the patient's attempted suicide, he received psychological treatment after discharge and seemed to have fully recovered at his 3-month follow-up. Both pharmacological and cognitive-behavioral therapies were very important for this patient. It is easy to ignore the importance of psychological factors in suicide for clinicians. Therefore, as clinicians, we must pay great attention to the psychological factors to avoid future incidences.

## CONCLUSION

In conclusion, acute sodium ferrocyanide and methanol poisoning can be successfully treated with plasmapheresis and CRRT. For an emergency physician, blood purification is an effective method to scavenge toxicants from the body in acute poisoning. The application of extracorporeal modalities including plasmapheresis and CRRT requires a thorough knowledge of drug pharmacokinetics, including the techniques utilized, and comprehensive analysis of the disease.
